# Interaction of a Preventative Fungicide Treatment and Root Rot Pathogen on Ambrosia Beetle Attacks during a Simulated Flood Event

**DOI:** 10.3390/insects9030083

**Published:** 2018-07-14

**Authors:** Karla Addesso, Fulya Baysal-Gurel, Jason Oliver, Christopher Ranger, Paul O’Neal

**Affiliations:** 1 Otis L. Floyd Nursery Research Center, Department of Agricultural and Environmental Sciences, Tennessee State University, McMinnville, TN 37110, USA; fbaysalg@tnstate.edu (F.B.-G.); JOliver@tnstate.edu (J.O.); poneal@blomand.net (P.O.); 2 Horticultural Insects Research Lab, Application Technology Research Unit, USDA-Agricultural Research Service, Wooster, OH 44691, USA; Christopher.Ranger@ARS.USDA.GOV

**Keywords:** granulate ambrosia beetle, *Xylosandrus*, *Phytophthora*, pyraclostrobin, boscalid, flood stress

## Abstract

Flooding can increase tree susceptibility to root rot pathogens as well as attacks by ambrosia beetles attracted to stress-induced ethanol emissions. The objective of this study was to investigate the interaction of a preventative fungicide treatment and root infection with *Phytophthora cinnamomi* on ambrosia beetle attacks in flood stressed trees. A fungicide (Pageant^®^ Intrinsic^®^) was evaluated in two flood trials using Eastern redbud and tulip poplar trees with treatments including the fungicide with or without pathogen or no fungicide with or without pathogen. Fungicide treated trees had fewer ambrosia beetle attacks, particularly in trees without *P. cinnamomi* co-infection. In a follow-up experiment, ethanol content was evaluated in flooded redbuds to determine if the fungicide treatment reduced stress-induced compounds. All flood stressed trees began producing ethanol within 24 h post flooding, regardless of fungicide treatment or *P. cinnamomi* infection. We conclude that pre-treatments of a fungicide can provide protection from ambrosia beetle attacks during an extreme flood event, but that protection is reduced if a root rot pathogen is also present. Additionally, rejection of fungicide treated trees was not related to the absence of ethanol, as the fungicide-treated plants released ethanol in quantities similar to non-treated trees.

## 1. Introduction

Plants in nurseries are often exposed to multiple biotic and abiotic stresses simultaneously, but research and management strategies usually focus on individual crop–pest interactions. Environmental stress, particularly flooding, predisposes trees to invasion by borers and root diseases, which often results in tree mortality [1]. Recently, nurseries in coastal areas of the southeastern US experienced major flooding during Hurricane Matthew [2] and other unusual spring storm events. Natural disasters are unpredictable, and nurseries have difficulty preparing for these events. Afterwards, nursery producers require management recommendations to mitigate the impacts of unexpected flood events on the woody ornamental crop. Management strategies that address multiple abiotic and biotic stressors and their interactions will provide better management recommendations for nursery crop pests and diseases than strategies that focus on only one stressor at a time.

Flooding of woody ornamental crops in field soil or container media poses two major problems in nursery production: it causes direct stress to the plant by depriving the roots of oxygen and it provides a vehicle for spread of soil- and water-borne oomycete pathogens among hosts. Environmental stress predisposes trees to pathogen infection by reducing host defenses [1]. In particular, flood stress can reduce root health, while increasing dispersal of zoospores in the soil [3,4]. Flooding creates anoxic conditions in the soil, which induces anaerobic respiration in the roots and ethanol as a by-product [3]. Ethanol produced in the roots is transported to aboveground portions of the tree, where some is released to the atmosphere [3,5,6]. Ethanol is then utilized as a kairomone by a suite of native and exotic ambrosia beetles to locate stressed host plants for colonization [7,8,9]. Adult female ambrosia beetles bore into trees, creating galleries (i.e., tunnels) in the vascular tissue [10]. As the beetle moves through the galleries, its obligate symbiotic fungus is inoculated on the walls of the galleries [11]. The presence of ethanol within tree tissues subsequently benefits their fungus farming by promoting the growth of the fungal symbiont, while inhibiting the growth of other competing microorganisms [12]. After fungal colonization, the parent female beetle deposits eggs in the galleries [11]. The larvae and adults must then consume the fungal symbiont in order to properly develop and reproduce [11]. Beetles overwinter in the adult stage and emerge in the spring, when trees are breaking dormancy, to search for a new host [13].

Both ambrosia beetles and *Phytophthora* root rot are recognized as management challenges for woody ornamental nursery production, but they are usually investigated separately, despite the fact that the same factor—flooding—is often responsible for both problems. Individual trees vary in their ability to tolerate flood stress and emit ethanol as a result of that stress [3,14,15]. Higher stress-related volatile emissions occur when flood intolerant species are flooded [5,6,15,16,17]. Likewise, tree species and cultivars may vary in their susceptibility to species of *Phytophthora* and that susceptibility can increase with the duration of flood stress. In eight species of ornamental *Prunus*, a combination treatment of flooding and inoculation with *Phytophthora cryptogea* Pethybr. & Laff. increased disease severity compared to flooding or inoculation alone [18]. Also, disease severity increased at longer flood durations in some cultivars of *Rhododendron* when inoculated with *Phytophthora cinnamomi* Rands [19].

Soil-borne pathogens like *P*. *cinnamomi* can cause major losses in nursery production [20]. Many popular ornamental genera are affected by various species of *Phytophthora* including *Acer*, *Cercis*, *Cornus*, *Juglans*, *Liriodendron*, *Magnolia*, *Prunus*, *Rhododendron*, and *Quercus* [20,21]. The host range of *Phytophthora* overlaps broadly with the host range of key ambrosia beetle pests [7,17]. Both granulate ambrosia beetle (*Xylosandrus crassiusculus* (Motschulsky)) and black stem borer) *Xylosandrus. germanus* (Blandford)) have a wide host range of over 100−200 species of mostly deciduous trees and shrubs [22]. Due to their propensity to attack living trees, the granulate ambrosia beetle and black stem borer are the most important ambrosia beetle pest species in several ornamental nursery regions in the United Stated [12,16,23,24]. Other invasive species like *Anisandrus maiche* Stark, camphor shot borer (*Cnestus mutilatus* (Blandford)), and polyphagous shot hole borer (*Euwallaecea* sp.), could become problematic nursery pests as they spread to nursery production regions [25,26,27].

Both *Phytophthora* root rot infection and ambrosia beetle attacks can result in tree decline or death [20,28]. Symptoms of *Phytophthora* root rot include small, yellow, wilted foliage and limb dieback [20]. Symptoms of ambrosia beetle attack include sawdust toothpicks extending from entry holes, leaking sap, dieback, wilting or yellowing foliage, and discolored bark [15,22]. Best practices for managing both *Phytophthora* infection and ambrosia beetle attacks includes minimizing abiotic stressors, including flooding. Growers are encouraged to keep fields graded and container yards well drained [17,20,29]. Optimal drainage is not always possible when unexpected weather events like heavy rains in the spring raise water content of soil and container media above recommended levels [17].

Both ambrosia beetles and *Phytophthora* root rot are easier to prevent than control, and even chemical management recommendations are weighted heavily on the preventative side. Management of ambrosia beetles can be difficult due to the beetles’ cryptic lifestyle inside the tree host, which limits exposure to insecticides. Additionally, the beetles do not feed on the tree host, so systemic insecticides evaluated have been ineffective [30]. As a result, trunk applications of insecticide are timed closely with the onset of ambrosia beetle attacks [13]. Current ambrosia beetle management recommendations in woody ornamental nurseries include monitoring for ambrosia beetle emergence in the spring with ethanol-baited traps and repeated preventative applications of pyrethroid insecticides once adult ambrosia beetles are detected [30,31].

The strobilurins, one fungicide class used to prevent Phytophthora root rot, act directly on the pathogen through the inhibition of mitochondrial respiration in plant pathogenic fungi/oomycetes [32]. In addition to direct action, these fungicides induce physiological changes in plants which can improve plant growth, increase stress tolerance, and induce disease resistance in the host [33,34,35,36]. The mechanism of stress tolerance in plants treated with strobilurins is unclear, but changes in phytohormone levels, especially decreased ethylene biosynthesis, and reduced free radical production caused by increased antioxidative enzymatic activity are at least partly responsible [37,38,39]. The strobilurins may provide indirect fungicidal activity by priming the host’s immune response to future attack by pathogens [33]. Priming is a phenomenon which causes an enhanced and quicker response upon future exposure to biotic or abiotic stress. The primed state can be induced by prior pathogen exposure, chemical agents, or beneficial soil microbes [39].

Some strobilurin fungicides have shown efficacy against ambrosia beetles through their effects on host suitability to the beetles and/or their symbiotic fungi. Preventative fungicide treatment with azoxystrobin had variable efficacy in reducing ambrosia beetle attacks, but did reduce the number of galleries with symbiont fungal growth compared to non-treated trees [31]. Pyraclostrobin was found effective in vitro against laurel wilt, *Raffaelea lauricola* T. C. Harr., Fraedrich and Aghayeva, the symbiotic fungus of *Xyleborus glabratus* Eichoff [40]. Due to their purported “stress-mitigating” properties, strobilurins may also act to suppress ambrosia beetle attacks indirectly if those treatments moderate tree stress signals used by the beetles to locate and select suitable hosts.

In the last two years, several major flood events across Southeastern Unites States nursery production regions have raised questions about how the interactions of pathogens and disease treatments may mitigate ambrosia beetle attacks. The main aim of this work was to investigate the interaction of a preventative fungicide treatment and a root rot pathogen on ambrosia beetle attacks under a simulated flood event. The three questions we sought to answer were: (1) do preventative fungicide treatments reduce ambrosia beetle attacks during a subsequent flood event; (2) are flooded trees co-infected with *Phytophthora* more likely to be attacked by ambrosia beetles; and (3) do fungicide treatments reduce ethanol emissions by mitigating the tree stress response?

## 2. Materials and Methods

### 2.1. Plant Material

Eastern redbud (*Cercis canadensis* L.) and tulip poplar (*Liriodendron tulipifera* L.) bare root liners were transplanted into #3 (11.4 L, C1200, Hummert International, Earth City, MO, USA) containers containing Pro-Gro Mix (Barky Beaver, Moss, TN, USA; 78% pine bark, 12% peat moss, 10% sand, and 4.8 kg lime/m^3^ with a manufacturer reported bulk density range of 240.3 to 256.3 kg/m^3^) amended with fertilizer (18-6-12 Osmocote fertilizer with micronutrients, ICL Fertilizers Company, Dublin, OH, USA) and maintained with overhead irrigation. Plants in Trial 1 were transplanted in April 2015 and evaluated in May 2016. Plants in Trial 2 were transplanted in February 2016 and evaluated in June 2016.

### 2.2. Field Trials

#### 2.2.1. Trial 1

Twenty redbud and twenty tulip poplar trees were randomly assigned to the following four treatments: (1) untreated check (fungicide−/inoculation−), (2) fungicide (fungicide+/inoculation−), (3) inoculated (fungicide−/inoculation+), and (4) fungicide and inoculated (fungicide+/inoculation+). Five reps of redbud and 5 reps of tulip poplar were assigned to each treatment. On 15 April 2016, trees in the fungicide treatment were pre-treated with 473 mL (16 fl oz) of Pageant^®^ Intrinsic^®^ fungicide solution (a.i. 12.8% pyraclostrobin and 25.2% boscalid; BASF Corporation, Research Triangle Park, NC, USA) at a rate of 1.35 g/L (18 oz/100 gallons). Pageant^®^ Intrinsic^®^ fungicide was applied as a “sprench” to the lower trunk and soil surrounding the base of the tree. Trees were lightly watered the following days to ensure maximal exposure of product. On 21 April, six days after fungicide application, plants were inoculated with *P*. *cinnamomi* grown on rice grains for 10 days only in inoculated designated treatments. Four rice grains were placed 1 cm (0.4 in) below the surface of the pine bark substrate, one in each of the four quadrants of the container. Plants were held for an additional 6 days to allow *P. cinnamomi* to establish in the pine bark media. On 27 April, trees were placed in 18 L buckets with plastic bag liners and flooded to above the root crown. On 4 May, plants were treated again with Pageant^®^ Intrinsic^®^ only in fungicide designated treatments and deployed along a tree line. Ambrosia beetle attacks were recorded on 6, 9, 11, 13, 16, 18, and 20 May. On 20 May, tree trunks were dissected for ambrosia beetle activity, and the container substrate was washed from the tree roots and roots were evaluated for root rot disease severity. The total number of ambrosia beetle attacks, percent of attacks resulting in gallery formation, and the percent of galleries with eggs were recorded. Root health scores were as follows: 1 = healthy roots, 2 = 1–25%, 3 = 26–50%, 4 = 51–75%, and 5 = 75–100% diseased roots. Average maximum temperatures for 15 April to 20 May were 22–27 °C (and 73–81 °F); average minimum temperatures were 8–10 °C (48–49 °F); and total rainfall was 6.53 cm (2.57 in).

#### 2.2.2. Trial 2

On 25 May, redbud and tulip poplar trees in the fungicide treatment were pre-treated as in Trial 1. On 1 June 2016, seven days after fungicide application, plants were inoculated with *P. cinnamomi* only in inoculated designated treatments. On 3 June, trees were placed in 18 L buckets with plastic bag liners and flooded to above the root crown. On 10 June, plants were treated again with Pageant^®^ Intrinsic^®^ only in fungicide designated treatments and deployed along a tree line. Ambrosia beetle attacks were recorded on 13, 15, 17, 20, 22, and 24 June. On 24 June, tree trunks were dissected for ambrosia beetle activity, and roots were evaluated for root rot disease severity as described above. Average maximum temperatures for 31 May and 24 June were 30–32°C (87–89 °F); average minimum temperatures were 15–16 °C (59–61 °F); and total rainfall 11.9 cm (4.69 in).

### 2.3. Evaluation of Ethanol Emission from Fungicide Treated Redbuds

Ethanol content was measured using Solid Phase Microextraction-Gas Chromatography-Mass Spectrocopy (SPME-GC-MS) [26] to determine if differences in ethanol emissions could explain the observed pattern of ambrosia beetle attacks. Redbud trees in #3 containers were treated as previously described. Bark tissue was removed from 4 reps of each treatment using a cork borer (0.5 cm dia) at 1, 3, and 7 days post flooding. Samples were placed in a sealed 4 mL vial and held on ice for transport to the lab. Bark cores were removed from ice and allowed to equilibrate at room temperature for 30 min. The SPME fibers (75 μm PDMS/Carboxen fiber, Sigma–Aldrich, St. Louis, MO, USA) were exposed to the headspace in the vial for 30 s. The SPME fiber samples were analyzed on GC-MS by manual injection on a Shimadzu QP-2010 GC-MS (Shimadzu Corp., Kyoto, Japan) fitted with a Merlin seal injection port (Sigma–Aldrich, St. Louis, MO, USA) and SPME liner and run on splitless mode. The injection port was held at 220 °C and the sample run on a DB-1ms column (25 µm, 0.25 mm, 30 m; Agilent Technologies, Santa Clara, CA, USA) from 35 °C to 100 °C. The ethanol peak was identified and area under the curve calculated.

### 2.4. Data Analysis

Root health scores, ethanol values, and the percentage of gallery formation were analyzed using a generalized linear model fitted to normal distribution (PROC GENMOD, SAS 9.3, SAS Institute, Inc., Cary, NC, USA). Ambrosia beetle hits and the percentage of galleries with eggs were analyzed using a generalized linear model fitted to a negative binomial distribution with a log link (PROC GENMOD, SAS Institute, Inc., Cary, NC, USA). Pairwise comparisons were made using LSmeans with a Scheffe adjustment to account for multiple comparisons.

## 3. Results

### 3.1. Root Health Scores

**Figure 1:**
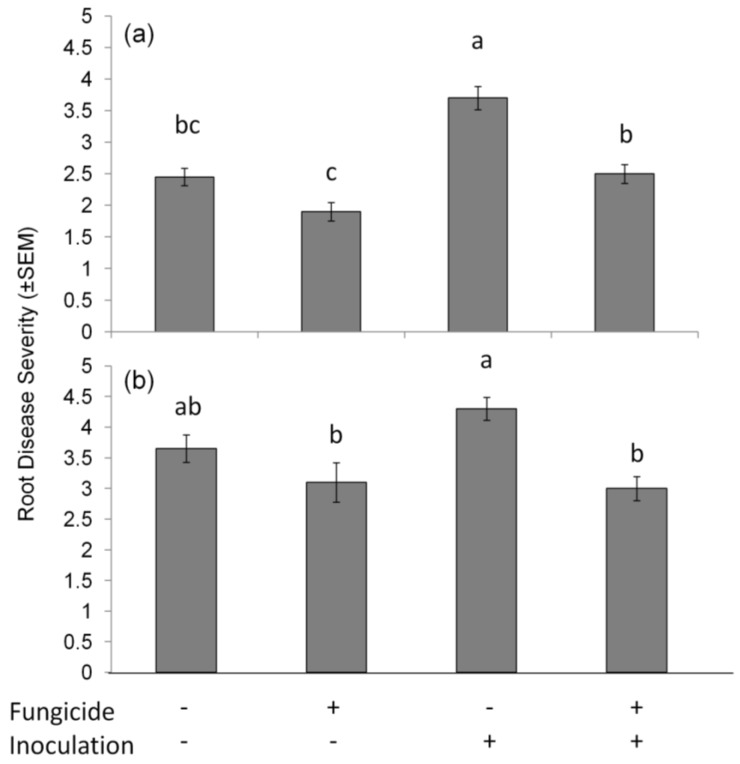
Root disease severity (±SEM) of flooded trees in (**a**) Trial 1; and (**b**) Trial 2. Fungicide+ = plants treated with Pageant^®^ Intrinsic^®^; Inoculation+ = plants inoculated with *P. cinnamomi*. Tree data in both trials represents combined redbud and tulip poplar data, since no differences were detected between tree species. Bars with different letters are significant at *p* < 0.05 by Scheffe pair-wise comparison.

Redbud and tulip poplar trees were evaluated for root health following a simulated flood event to assess disease severity across treatments. Trials were analyzed separately due to differences between root health response in Trials 1 and 2. There were no differences detected between the tree species in Trial 1 (F_1,36_ = 3.02, *p* = 0.091). Root damage in Trial 1 was greater in trees inoculated with *P. cinnamomi* (F_1,36_ = 34.13, *p* < 0.000; Figure 1a) and those that were not treated with Pageant^®^ Intrinsic^®^ fungicide (F_1,36_ = 30.54, *p* < 0.000). In Trial 2, there were again no differences detected between the tree species (F_1,36_ = 0.01, *p* = 0.920). Root damage in Trial 2 was not affected by the inoculation of *P. cinnamomi* (F_1,36_ = 1.24, *p* = 0.273; Figure 1b); however, those plants treated with fungicide had less root damage (F_1,36_ = 30.54, *p* < 0.000). Interaction terms were not significant and subsequently removed from the models.

### 3.2. Ambrosia Beetle Attacks

**Figure 2:**
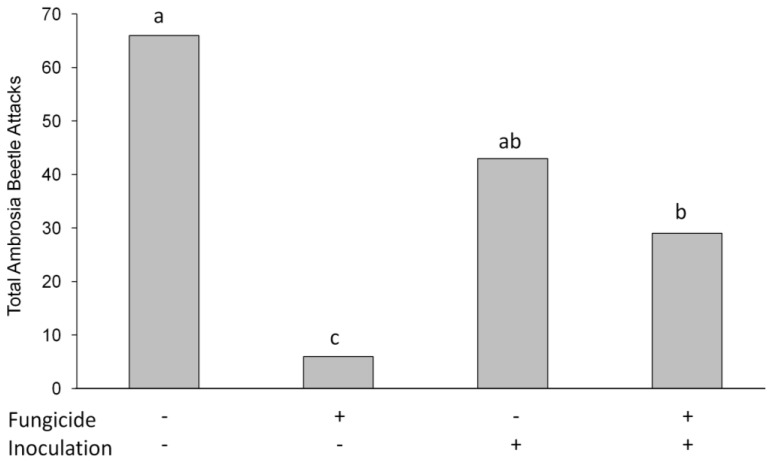
Total ambrosia beetle attacks on flooded redbud and tulip poplar trees (tree species data were combined due to no statistical difference detected). Data also represent combined Trial 1 and 2 data due to low ambrosia beetle incidence in trials. Fungicide+ = plants treated with Pageant^®^ Intrinsic^®^; Inoculation+ = plants inoculated with *P. cinnamomi*. Bars with different letters are significant at *p* < 0.05 by Scheffe pair-wise comparison.

Due to low total ambrosia beetle attacks, the two trials were analyzed together. There were no differences between trials (F_1,74_ = 2.10, *p* = 0.15) A significant fungicide by inoculation interaction was observed (F_1,74_ = 4.80, *p* = 0.03). The fungicide treatments were different (F_1,74_ = 11.43, *p* = 0.001; Figure 2). Fewer ambrosia beetle attacks were observed on Pageant^®^ Intrinsic^®^ treated trees, but the pattern was much stronger in the non-inoculated trees. Inoculation (F_1,75_ = 2.9, *p* = 0.09) and tree species treatments (F_1,75_ = 3.29, *p* = 0.07) were not different. The suite of ambrosia beetles attacking the trees included granulate ambrosia beetle, camphor shot borer and black stem borer.

### 3.3. Ambrosia Beetle Attacks Resulting in Gallery Formation

**Figure 3:**
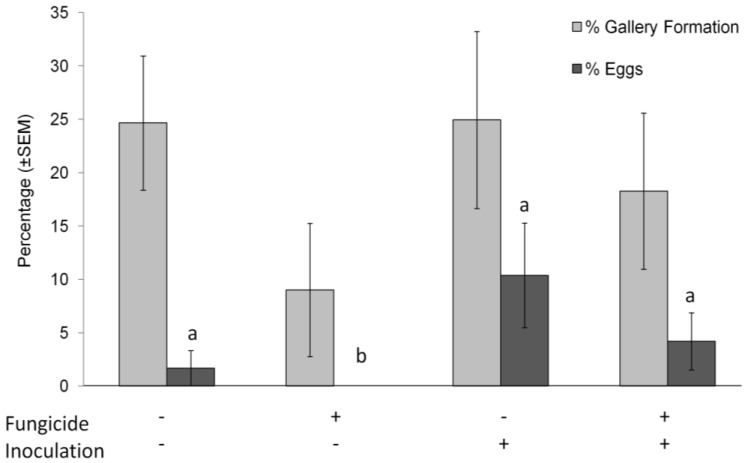
Post-attack successful ambrosia beetle % gallery formation (±SEM) and % of galleries containing eggs (±SEM). Fungicide+ = plants treated with Pageant^®^ Intrinsic^®^; Inoculation+ = plants inoculated with *P. cinnamomi*. Bars with different letters are significant at *p* < 0.05 by Scheffe pair-wise comparison.

No differences were observed in percent of ambrosia beetle attacks resulting in gallery formation between Trials 1 and 2 (F_1,74_ = 1.86, *p* = 0.172); therefore, data were analyzed together. Interaction terms were not significant and subsequently removed from the model. No differences were observed in percent of successful gallery formation between tree species (F_1,74_ = 1.67, *p* = 0.196; Figure 3), fungicide application (F_1,74_ = 2.04, *p* = 0.153) or *P. cinnamomi* inoculation treatments (F_1,74_ = 1.08, *p* = 0.298). In short, if ambrosia beetles attacked a tree, gallery formation followed at the same level across all treatments.

### 3.4. Ambrosia Beetle Galleries with Eggs

No differences in the egg load were observed between Trials 1 and 2 (F_1,74_ = 0.39, *p* = 0.532) and data were combined for analysis. Interaction terms were not significant and subsequently removed from the model. Differences in egg load were observed between the two tree species (F_1,74_ = 4.84, *p* = 0.028; Figure 3), with more eggs laid in tulip poplar than redbud. A difference was observed between *P. cinnamomi* inoculated and non-inoculated treatments (F_1,74_ = 11.73, *p* = 0.001), with more eggs laid in *P. cinnamomi* inoculated trees. Fungicide treatments were also different (F_1,74_ = 10.31, *p* = 0.001), with fewer eggs laid in the Pageant^®^ Intrinsic^®^ treated trees without inoculation.

### 3.5. Evaluation of Ethanol Emission from Fungicide Treated Redbuds

**Figure 4:**
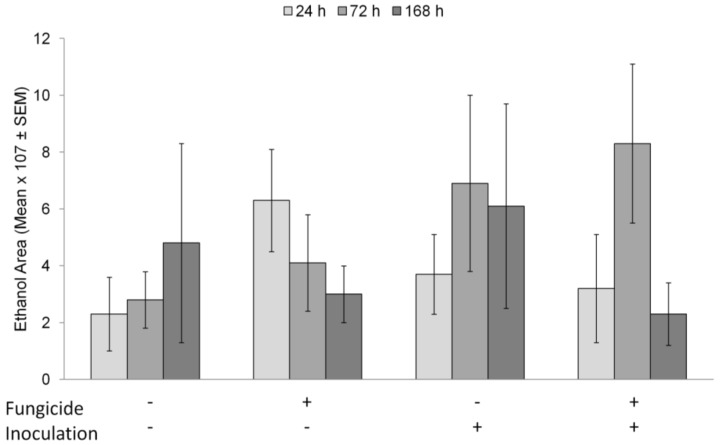
Ethanol emission (±SEM) from trees at 24, 72, and 168 h of flooding. Fungicide+ = plants treated with Pageant^®^ Intrinsic^®^; Inoculation+ = plants inoculated with *P. cinnamomi*. Bars with different letters are significant at *p* < 0.05 by Scheffe pair-wise comparison.

All redbud treatments subjected to flooding began releasing ethanol by 24 h post flooding. Ethanol release from the individual trees was variable, but no differences were observed in ethanol emissions by day (*F*_2,43_ = 0.64, *p* = 0.532), *P. cinnamomi* inoculation (F_1,43_ = 0.89, *p* = 0.350) or fungicide treatment (F_1,43_ = 0.00, *p* = 0.955) (Figure 4).

## 4. Discussion

In nursery production regions prone to flood events, the dangers to trees are two-fold: direct stress to the plant due to apoxic conditions in the root zone [3] and exposure to water dispersed pathogens such as *Phytophthora* [3,4]. The former condition is known to lead to anaerobic respiration and release of ethanol by the trees. The ethanol stress signal can induce subsequent ambrosia beetle attacks [7,15,16]. While most nursery management studies treat ambrosia beetle and *Phytophthora* management separately, flood events can cause the two problems to co-occur. If a nursery has a history of problems with *Phytophthora* infection, the producers may treat plants preventatively for root rot. Fungicides in several classes are available for preventative treatments, but one class of products, the strobilurins, have been reported in other studies to mitigate stress, promote growth, and reduce damage by pests and pathogens [31,41]. 

In this study, we treated redbud and tulip poplar plants preventatively at the recommended 14 d interval for root rot with a commercially available fungicide Pageant^®^ Intrinsic^®^, just as a nursery producer might do prior to a flood event. We knew the fungicide would have some protective effect against *P. cinnamomi*, and this was confirmed by our root disease severity ratings (Figure 1). We also hypothesized that the fungicide would reduce ambrosia beetle attacks, which it did, although the strength of that protection was greater in the non-inoculated treatments and reduced in trees inoculated with *P. cinnamomi* (Figure 2). While the differences were not significant, there were fewer total ambrosia beetle hits on *P. cinnamomi* inoculated trees (*n* = 43) compared to non-inoculated trees that were flood stressed (*n* = 66). Based on these results, we conclude that the preventative fungicide treatment reduced both *P. cinnamomi* disease severity and ambrosia beetle attacks following a simulated flood event. 

Previous studies in oak trees have reported attraction of ambrosia and related bark beetle species to trees exhibiting cankers from sudden oak death, *Phytophthora ramorum* (S. Werres & A. W. A. M. de Cock) [14,41], so attraction of ambrosia beetles to *Phytophthora* infected plants is not without precedent. Kelsey et al. [14] determined that emission of ethanol from canker tissue was responsible for the attraction. Therefore, it is reasonable to predict that if *Phytophthora* infection progresses far enough to inhibit respiration, ambrosia beetle attacks will decline with the declining ethanol signal. Further investigations with different inoculum levels of *Phytophthora* may clarify whether there is a disease incidence at which ambrosia beetles will begin to reject host trees due to a perceived disadvantage to their symbionts.

Why the fungicide treatment alone reduced ambrosia beetle attacks is unclear. Our first hypothesis was that fungicide treated plants had reduced ethanol emissions and were therefore less attractive to the beetles. The subsequent ethanol emission experiment suggests that this is not the case, as all treatments released ethanol within 24 h of flooding (Figure 4). Based on the ethanol emission data, we conclude that another signal, aside from a lack of ethanol, is responsible for ambrosia beetle rejection of fungicide treated plants. 

The gallery formation and egg deposition data does suggest that fungicide-treated-trees are less suitable hosts. The variation among treatments requires caution when interpreting the gallery formation data, but what is clear is that no eggs were laid in non-inoculated, fungicide treated plants during the two trial periods. Tpercent of eggs laid in the non-inoculated treatments was less than the combined *Phytophthora* inoculated treatments, so it is also possible *Phytophthora* presence may be conditioning the trees to be more suitable for colonization, even when fungicide is added. If the beetles rejected the fungicide treated trees because they were unsuitable for symbiont establishment, some signal other than ethanol is likely involved. 

In a previous study, the fungicides azoxystrobin and potassium phosphite did not reduce ambrosia beetle attacks on flood-stressed trees, but did reduce the number of galleries in the host trees containing fungal colonies [31]. Furthermore, galleries containing eggs were detected in the non-treated trees, but not in trees preventively treated with azoxystrobin or potassium phosphite. Thus, pre-treatment with azoxystrobin and potassium phosphite reduced the colonization success of ambrosia beetles since oviposition is not initiated until the fungal colonies are established. Since few attacks were initiated in the fungicide treatment (*n* = 6), we predict that the signal causing rejection of Pageant treated trees was detected prior to or upon landing on the plant.

When and why the beetles rejected the fungicide treated trees remains to be determined. Current behavioral experiments to address this question include observations of beetle landing, host exploration, and gallery initiation on fungicide treated and non-treated flood stressed trees. These trials are intended to determine whether the signal responsible for rejection of the host is a long-range volatile, and/or a contact or gustatory stimulus. Upon completion of behavioral trials, isolation and identification of compounds responsible for rejection behavior may be investigated.

## 5. Conclusions

We conclude that a preventative application of Pageant fungicide can reduce ambrosia beetle attacks and root rot damage during a subsequent flood event. Our results also demonstrate that trees inoculated with *P*. *cinnamomi* are not more likely to be attacked by the beetles than non-infected, flood stressed trees. All tree treatments emitted ethanol, so a signal other than absence of the ambrosia beetle primary attractant caused rejection of the trees.
